# Updating Categorical Soil Maps Using Limited Survey Data by Bayesian Markov Chain Cosimulation

**DOI:** 10.1155/2013/587284

**Published:** 2013-08-20

**Authors:** Weidong Li, Chuanrong Zhang, Dipak K. Dey, Michael R. Willig

**Affiliations:** ^1^Department of Geography and Center for Environmental Sciences & Engineering, University of Connecticut, Storrs, CT 06269, USA; ^2^Department of Statistics, University of Connecticut, Storrs, CT 06269, USA; ^3^Center for Environmental Sciences & Engineering and Department of Ecology & Evolutionary Biology, University of Connecticut, Storrs, CT 06269, USA

## Abstract

Updating categorical soil maps is necessary for providing current, higher-quality soil data to agricultural and environmental management but may not require a costly thorough field survey because latest legacy maps may only need limited corrections. This study suggests a Markov chain random field (MCRF) sequential cosimulation (Co-MCSS) method for updating categorical soil maps using limited survey data provided that qualified legacy maps are available. A case study using synthetic data demonstrates that Co-MCSS can appreciably improve simulation accuracy of soil types with both contributions from a legacy map and limited sample data. The method indicates the following characteristics: (1) if a soil type indicates no change in an update survey or it has been reclassified into another type that similarly evinces no change, it will be simply reproduced in the updated map; (2) if a soil type has changes in some places, it will be simulated with uncertainty quantified by occurrence probability maps; (3) if a soil type has no change in an area but evinces changes in other distant areas, it still can be captured in the area with unobvious uncertainty. We concluded that Co-MCSS might be a practical method for updating categorical soil maps with limited survey data.

## 1. Introduction

Soil is an important natural resource and is also an essential component of ecosystems. The spatial distribution of different soils represents a special kind of natural landscapes (called soilscape). Soils are traditionally classified into a number of types and delineated as categorical maps based on multiple attributes observed at sample profiles, tacit knowledge of experienced surveyors, remotely sensed landscape features, and a specific classification system. Categorical soil maps are widely used in ecological and agricultural studies and provide crucial information for natural resource and environmental management. Because existing soil maps may be of low quality or too outdated to reflect current soil distributions, map update is necessary for providing current, more accurate, or more detailed information to meet the requirements of applications. For example, most soil series maps in United States (e.g., the USDA Soil Survey Geographic Database) were made on the basis of field surveys carried out in the 1950s, and they may not have been effectively updated to reflect recent soil changes. However, large-scale detailed soil survey is too costly to be carried out frequently for generating new high-quality maps. If an existing soil map is of sufficient quality and appropriately scaled, updating may not require a new full-coverage soil survey for a revised soil map because the types of soils at most places in the legacy map may not have changed. Consequently, we may be able to update a legacy soil map with only limited new survey data on soil distribution. When qualified legacy soil maps are available, we may only need to address areas where the previously determined soil types have a large possibility of type change due to some reasons (e.g., internal or environmental changes, incorrect mapping, or taxonomy change), identified by careful map examination with ancillary information. Changes can be found through a limited soil update survey or simply map examination by experts. Other reasons of using legacy soil maps and survey data together to create current categorical soil maps include that: (1) historical field survey data were not well kept or were kept without accurate coordinates and (2) legacy soil maps were based on drawings of experienced soil surveyors during field surveys, but most observed soil profiles were not sampled for laboratory analysis or recorded into a database. In general, we may incorporate information from a legacy soil map into the current soil map based on limited survey data if the legacy soil map contains valuable information that cannot be replaced by a limited survey.

A variety of quantitative modeling methods have been used or developed to predict spatially explicit soil categorical characteristics. These methods may have their own merits in different contexts. One group of methods is soil-landscape models, which use environmental soil-forming factors to predict soil patterns over unvisited areas. These methods include multinomial logistic regressions (MLRS), classification and regression tree analysis, and fuzzy methods; see applications in predictive categorical soil mapping [1–9]. This group of methods generally does not incorporate spatial autocorrelations. The other group considers spatial statistical models, mainly including indicator geostatistics, maximum entropy models, and Markov chain random fields (MCRF); see related studies [10–12] in mapping categorical soil variables. These methods are based on spatial autocorrelations of categories, but legacy data and remotely sensed landscape data may also be incorporated as auxiliary information. Other spatial statistical methods that were suggested for mapping categorical variables may also be used or adapted for mapping soil categories [13, 14]. In addition, some qualitative methods such as the rule-based method [15] and the pure remote sensing method [16] were introduced recently for mapping soil types, but only for special soil types such as peat lands or gypsic soils.

Recently, Markov chains were extended into a new spatial statistical approach, that is, the MCRF approach, for simulating categorical spatial variables [17]. This approach uses transiograms [18] to measure class spatial auto- and cross-correlations and uses MCRF models (usually simplified models) to estimate the local conditional probability distribution of a categorical spatial variable at an unobserved location. MCRFs may be regarded as an extension of Markov mesh random fields [19] toward conditional simulation on sample data or as a special kind of causal Markov random fields in accordance with the Bayesian inference principle. MCRF-based sequential simulation algorithms can be used to generate simulated realizations in single sweeps, similar to other geostatistical sequential simulation algorithms. This approach may incorporate various interclass relationships, thus effectively reducing the uncertainty associated with prediction and generating more accurate simulated realizations that strictly obey class neighboring relationships [20]. Nonetheless, currently implemented MCRF algorithms do not incorporate auxiliary or legacy data by cosimulations, thus requiring further extensions. 

It is easy to understand that legacy soil data, whether they are map data or observed point data, contain valuable information that is relevant to present soil patterns. Legacy soil maps also contain the tacit knowledge of experienced surveyors, who were intensively trained for soil survey but may not be available at the time of soil map updating [21]. Therefore, proper use of legacy soil data may appreciably improve the prediction of soil spatial distributions. In fact, the use of legacy soil data in digital soil modeling has become a commonplace [22]. If densely distributed survey data are not available, a legacy soil map available at a similar scale may be used as auxiliary data to create the current soil map with limited survey data. 

In this study, we assume that the legacy soil maps from the last update or made from last extensive soil surveys need limited corrections related to natural or anthropogenic soil changes or other reasons. Consequently, update is only necessary in altered areas or erroneously mapped locations. As such, we assume that the legacy soil maps are mainly outdated rather than being of low quality, and that update is necessary for a variety of reasons. This is reasonable because (1) many high-quality soil maps were made by extensive soil surveys, usually commissioned by government agencies, and (2) many soil types only change slowly as a result of natural processes, except for some special soil groups (e.g., hydric soils). Such an assumption may be applicable to many situations in the United States, where detailed large-scale categorical soil maps exist for each county in many states. To incorporate legacy soil maps through cosimulations for categorical soil map creation with limited survey data, the MCRF sequential simulation (MCSS) algorithm proposed in [20] was extended into a MCRF sequential cosimulation (Co-MCSS) algorithm and its workability was demonstrated by a case study on synthetic data in this study. The main objective is to suggest a suitable cost-efficient method for updating legacy categorical soil maps that only requires limited new survey data, mainly in the changed areas. It should be noted that although limited map changes in categorical soil map update may be carried out using a conventional hand-delineating method, a spatial statistical method would be appreciated due to many reasons, such as efficiency, objectivity in soil type boundary determination, and availability of uncertainty information associated with the updating. 

## 2. Methods

### 2.1. Markov Chain Random Fields

The chief obstacle to extending one-dimensional Markov chains to multidimensional causal random field models such as Markov mesh models [19] is the lack of a natural ordering for a multidimensional grid and hence the lack of a natural notion of causality in the spatial data. As a result, an artificial ordering for spatial data must be assumed, which often yields directional artifacts in simulated images [23, 24]. The MCRF theory solved this problem and other related issues that hindered conditional Markov chain simulations on sparse sample data. The initial ideas of MCRFs aimed to correct the flaws of a two-dimensional Markov chain model for subsurface characterization [17, 24]. The ideas were generalized into a theoretical framework for a new geostatistical approach for simulating categorical fields [17]. Wide applications of this approach lie within further extensions of MCRF models and the development of simulation algorithms that can effectively deal with data clustering (or redundancy), ancillary information, and multiple-point statistics. 

A MCRF refers to a random field defined by a single spatial Markov chain that moves or jumps in space and decides its state at any uninformed (i.e., unobserved and unvisited in a simulation process) location by interactions with its nearest neighbors in different directions and its last stay (i.e., visited) location [17]. The interactions within a neighborhood are performed through a sequential Bayesian updating process [25]. Therefore, a MCRF is a spatial Markov chain with local Bayesian updating. Here, a “state” means a category (or class) for a categorical spatial variable. For a MCRF *Z*(**u**), if we assume that *i*_1_ to *i*_*m*_ are the states of the nearest neighbors in different directions around an uninformed location **u**_0_ plus the state of the last visited location of the spatial Markov chain, the local conditional probability distribution of *Z*(**u**) at the current uninformed location **u**_0_ can be denoted as *p*[*i*_0_(**u**_0_) | *i*_1_(**u**_1_),…, *i*_*m*_(**u**_*m*_)], where *i*_0_ refers to the state of *z*(**u**_0_) being estimated. Emphasizing the single-chain nature of a MCRF and the last visited location, this local conditional probability distribution can be factorized as

(1) p[i0(u0) ∣ i1(u1),…,im(um)]  =1Ap[im(um) ∣ i0(u0),…,im−1(um−1)]   ⋯p[i2(u2) ∣ i0(u0),i1(u1)]p[i0(u0) ∣ i1(u1)],

where *A* = *p*[*i*_1_(**u**_1_),…, *i*_*m*_(**u**_*m*_)]/*p*[*i*_1_(**u**_1_)] is a normalizing constant and **u**_1_ indicates the last visited location or the location that the spatial Markov chain goes through to the current location **u**_0_ [17]. This explicit full general solution of MCRFs is essentially a multiple-point geostatistical model, composed of a series of two- to *m* + 1-point statistics (or cliques) involving the current uninformed location **u**_0_. These two- and multiple-point statistics are also functions of directional lag distances because these points are usually not immediately adjacent in a space of sample data. They may be estimated from training images but the computation is much complex. Note that the local joint probability distribution of *Z*(**u**); that is, *p*[*i*_0_(**u**_0_), *i*_1_(**u**_1_),…, *i*_*m*_(**u**_*m*_)] can be similarly factorized. 

If we consider (1) in the Bayesian inference formulation, *p*[*i*_0_(**u**_0_) | *i*_1_(**u**_1_),…, *i*_*m*_(**u**_*m*_)] is the posterior probability distribution, *p*[*i*_0_(**u**_0_) | *i*_1_(**u**_1_)] (i.e., a transition probability, or a transiogram if regarded as a function of the lag distance) is the prior probability distribution, and the other part of the right-hand side excluding the constant is the likelihood component. The prior probability indicates the single Markov chain nature of a MCRF. The likelihood component is composed of multiple terms (one for each nearest neighbor), which update the prior probability using nearest neighbors in different directions by a manner of recursion as follows:

(2) $$\begin{array}{c} \text{posterio}{\text{r}}_{1}=\text{prior}\\ \text{posterio}{\text{r}}_{2}\propto L_{2}\times \text{posterio}{\text{r}}_{1}\\ \cdots \\ \text{posterio}{\text{r}}_{m}\propto L_{m}\times \text{posterio}{\text{r}}_{m-1}\propto L_{m}\times \cdots \times L_{2}\times \text{prior}, \end{array}$$

where *L*_*k*_ refers to the likelihood term for the *k*th nearest neighbor, that is, *p*[*i*_*k*_(**u**_*k*_) | *i*_0_(**u**_0_),…, *i*_*k*−1_(**u**_*k*−1_)]. Thus, when no nearest neighbor other than the last visited location is available, we get a posterior probability equal to the prior probability (the likelihood term *L*_1_ is 1). But when there are nearest neighbors other than the last visited location available, update begins on each datum in turn, and in each time of update the posterior of last update serves as the new prior. Therefore, a MCRF model can be explained from the viewpoint of Bayesian inference. The generation of a MCRF may be regarded as a dynamic Bayesian inference process. Because the above Bayesian updating process is conducted simultaneously within a neighborhood rather than an iterative updating algorithm, it can be simply written as [25]

(3) posterior∝likelihood[im(um)] ×⋯×likelihood[i2(u2)]×prior.

**Figure 1:**
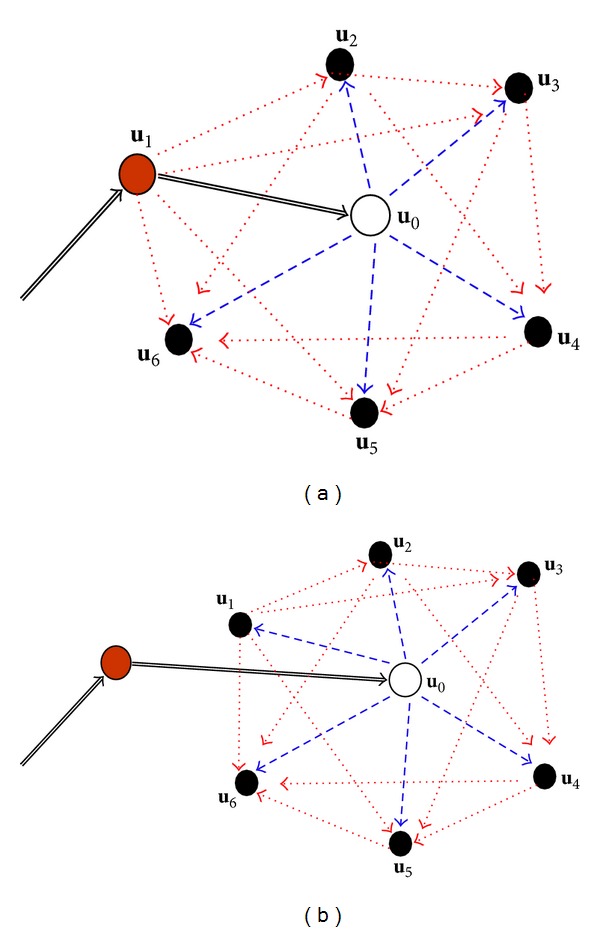
Neighborhood structures with six nearest neighbors and the sequential Bayesian updating process in basic Markov chain random fields: (a) assuming **u**_1_ to be the last visited location; (b) assuming the last visited location is far away (outside the neighborhood). Data interactions across the uninformed location **u**_0_ being estimated are ignored according to the Markov property.

This sequential Bayesian updating process on nearest neighbors starts from nearest neighbor *i*_2_(**u**_2_) and ends at nearest neighbor *i*_*m*_(**u**_*m*_) in a Markov-type neighborhood around the uninformed location **u**_0_ being estimated (see Figure 1(a) as an example). This updating process may not need to follow a fixed sequence of nearest neighbors because earlier considered nearest neighbors within the neighborhood become the conditioning data of later updates, and all updates are conditioned on the datum *i*_0_(**u**_0_) being estimated. Such a spatial estimation method is different from existing spatial estimation methods such as kriging and conventional Markov random field models.

If the spatial Markov chain is stationary and its last visited location is far away from the current uninformed location, the influence of the last visited location may be ignored (i.e., the transition probabilities from the last visited location to the current location decay to corresponding marginal probabilities). Thus, the local conditional probability distribution *p*[*i*_0_(**u**_0_) | *i*_1_(**u**_1_),…, *i*_*m*_(**u**_*m*_)] can be factorized differently as

(4) p[i0(u0) ∣ i1(u1),…,im(um)]  =1Ap[im(um) ∣ i0(u0),…,im−1(um−1)]   ⋯p[i1(u1) ∣ i0(u0)]p[i0(u0)],

where *A* = *p*[*i*_1_(**u**_1_),…, *i*_*m*_(**u**_*m*_)] is a normalizing constant and **u**_1_ is not the last visited location but just a nearest neighbor. Equation (4) is a special case of (1). If we consider this equation in the Bayesian inference formulation, *p*[*i*_0_(**u**_0_) | *i*_1_(**u**_1_),…, *i*_*m*_(**u**_*m*_)] is still the posterior, *p*[*i*_0_(**u**_0_)] (i.e., a marginal probability) becomes the prior, and the other part of the right-hand side excluding the constant is the likelihood component. For this special case, the sequential Bayesian updating process on nearest neighbors starts from nearest neighbor *i*_1_(**u**_1_) and ends at nearest neighbor *i*_*m*_(**u**_*m*_) in a Markov-type neighborhood around the location **u**_0_ being estimated (see Figure 1(b) as an example).

Because (1) involves complex multiple-point statistics that are difficult to estimate from sparse sample data, simplification is necessary. If we invoke the conditional independence assumption, a simplified general solution for MCRFs can be obtained from (1) as follows:

(5) p[i0(u0) ∣ i1(u1),…,im(um)]  =pi1i0(h10)∏g=2mpi0ig(h0g)∑f0=1n[pi1f0(h10)∏g=2mpf0ig(h0g)],

where *p*_*i*_0_*i*_*g*__(**h**_0*g*_) represents a transiogram (i.e., transition probability function) from class *i*_0_ at location **u**_0_ to class *i*_*g*_ at location **u**_*g*_ with the lag distance **h**_0*g*_; *i*_1_(**u**_1_) represents the nearest neighbor from or across which the spatial Markov chain moves to the current location **u**_0_; *m* represents the number of nearest neighbors plus the last visited location; *i* and *f* all represent states (i.e., classes) in the state space *S* = (1,…, *n*) of the categorical field under study. This simplified general solution is still in accordance with the Bayesian inference formulation. Because this simplified solution involves only two-point statistics—transiograms, which can be estimated from sample data, it is computable directly using sample data. In addition, because class proportions are not involved in this solution, no assumption is required concerning their stationarity. This simplified solution did not consider the data clustering issue and left it to model extension and specific algorithm design. Data clustering apparently impacts the contributions of nearest neighbors to the local conditional probability distribution at the current location being estimated, thus accounting for this effect is preferable when it is possible. For example, one may consider applying a set of power parameters to the transition probability terms based on the neighborhood configuration, but the computation load will inevitably largely increase.

If the spatial Markov chain is stationary and its last visited location is far from the current location **u**_0_ (i.e., outside the neighborhood), we have *p*_*i*_1_*i*__0_(**h**_10_) ≈ *p*_*i*_0__ due to Lim_**h**_10_→*∞*_ *p*_*i*_1_*i*_0__(**h**_10_) = *p*_*i*_0__, which is a basic property of one-dimensional first-order stationary Markov chains. Thus, if we still assume that there are *m* nearest neighbors, (5) may be rewritten as

(6) p[i0(u0) ∣ i1(u1),…,im(um)]  =pi0∏g=1mpi0ig(h0g)∑f0=1n[pf0∏g=1mpf0ig(h0g)],

where *p*_*i*_0__refers to the marginal probability of class *i*_0_, which is approximately equal to the mean value of the class proportion for omni- and bi-directional transition probabilities or a large study area. Equation (6) also can be obtained by simplifying (4) based on the conditional independence assumption or by transforming (5) using the relationship of *p*_*i*_0_*i*_1__(**h**_01_)*p*_*i*_0__/*p*_*i*_1__ = *p*_*i*_1_*i*_0__(**h**_10_). However, for spatial data, this relationship only holds in stationary situations and does not hold for nonstationary situations and unidirectional transiograms. Therefore, (6) is a special stationary case of (5) and is included in (5).

### 2.2. MCRF Cosimulation Model

To incorporate auxiliary variables, we need to expand (5) into a Co-MCRF model. The contributions of auxiliary variables may be incorporated by using the formulation of addition (to some extent similar to cokriging), that is, by including one contribution term for each auxiliary variable. Such a formulation must be renormalized or allocate weights to its contribution terms to ensure the total probability of occurrences of all states (i.e., classes) at location **u**_0_ sums to unity. Alternatively, the formulation of multiplication can be used to incorporate auxiliary variables. In this scenario, we regard the data of auxiliary variables as nearest neighbors of the uninformed location **u**_0_ in different variable spaces. Here, we use the multiplication formulation to construct the Co-MCRF model. We consider only the colocated cosimulation case because it is what we need for revising categorical soil maps while the involved auxiliary variables, for example, the legacy categorical soil map, provide exhaustive data. The colocated Co-MCRF model with *k* auxiliary variables can be written as

(7) p[i0(u0) ∣ i1(u1),…,im(um);r0(1)(u0);…;r0(k)(u0)]  =pi1i0(h10)∏g=2mpi0ig(h0g)∏l=1kbi0r0(l)∑f0=1n[pi1f0(h10)∏g=2mpf0ig(h0g)∏l=1kbf0r0(l)],

where *r*_0_^(*k*)^ represents the state of the *k*th auxiliary variable at the colocation **u**_0_. The cross-transiograms from the primary variable to auxiliary variables reduce to cross transition probabilities *b*_*i*_0_*r*_0__ due to the colocation property. We may call this kind of cross-transition probabilities (and transiograms) between classes of two different categorical fields *cross-field transition probabilities* (and *transiograms*). The cross-field transition probabilities, however, have to be estimated separately. In this equation, we do not deal with cross-correlations between auxiliary variables and practically consider them to be independent of each other.

In this study, we consider only one auxiliary variable in the form of a legacy soil map. Hence, (7) further reduces to

(8) p[i0(u0) ∣ i1(u1),…,im(um);r0(u0)]  =bi0r0pi1i0(h10)∏g=2mpi0ig(h0g)∑f0=1n[bf0r0pi1f0(h10)∏g=2mpf0ig(h0g)].

If an auxiliary variable has no correlation with the primary variable, the cross-field transition probabilities will equal the corresponding class mean proportions of the auxiliary variable, and the corresponding cross-field transition probability terms in (8) will be canceled from the numerator and denominator. 

### 2.3. MCRF Sequential Cosimulation Algorithm

The conditional independence assumption was assumed for nearest neighbors in different directions to derive the simplified general solution of MCRFs. Such an assumption is practical, often used in nonlinear probability models [26]. However, the conditional independence of adjacent neighbors in cardinal directions for a rectangular lattice is a property of Pickard random fields, a kind of unilateral Markov models [27–29]. For the situation of the four (or less) nearest neighbors found in cardinal directions, the conditional independence property of Pickard random fields may be applied to the sparse data situation [17, 24]. This supports the neighborhood choice of using four nearest neighbors in four cardinal directions or quadrants in MCRF algorithm design to reduce data clustering effects [20]. 

In fact, it is also unnecessary and difficult to consider many nearest neighbors in different directions in applications. Nearest neighbors outside correlation ranges can be eliminated from consideration. The influence of remotely located data on the current uninformed location is typically screened by closer data within a certain angle. In addition, the conditional independence assumption apparently does not hold for clustered sample data. Therefore, it is proper for MCRF-based Markov chain models to consider only the nearest neighbors in several cardinal directions within a search range to both approximately meet the conditional independence assumption and increase the computation efficiency. 

The four nearest neighbors in four cardinal directions can be regarded as conditionally independent given the state of the surrounded central location in a sparse data space [17]. Consequently, the neighborhood choice for the Co-MCRF model needs only to use the four nearest neighbors in four cardinal directions, allowing (8) to be further simplified to

(9) p[i0(u0) ∣ i1(u1),…,i4(u4);r0(u0)]  =bi0r0pi1i0(h10)∏g=24pi0ig(h0g)∑f0=1n[bf0r0pi1f0(h10)∏g=24pf0ig(h0g)].

**Figure 2:**
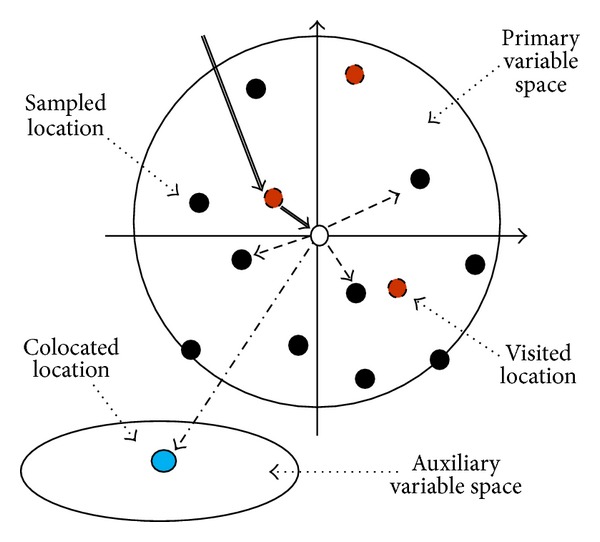
Illustration of the Markov chain random field colocated cosimulation model with quadrant search and one auxiliary variable for random-path sequential simulation. Double arrows represent the moving directions of the spatial Markov chain. Dashed arrows represent the interactions of the spatial Markov chain with nearest neighbors and auxiliary data.

Here, we assume that the last visited location of the spatial Markov chain is always within the four nearest neighbors; if it is not so, we assume that the spatial Markov chain comes through one of them (Figure 2). Such a simplified Co-MCRF model provides the MCRF approach the capability of dealing with large data sets.

A tolerance angle is required because nearest neighbors in a neighborhood may not be located exactly along cardinal directions. To cover the whole space of a search area, sectors can be substituted for cardinal directions, and we can seek one nearest neighbor from each sector to represent the neighborhood (Figure 2). If we consider four cardinal directions, the sectors representing cardinal directions are quadrants. There may be no data to occur in some quadrants within a search range at the boundary strips or at the beginning of a simulation when sample data are very sparse. Consequently, the size of a neighborhood may be less than four. Equation (9) can always be adapted to the situation. In case no data can be found in the whole search area, we assume the spatial Markov chain comes from a location outside the search range. By choosing a suitable search radius based on the density of sample data, this situation rarely occurs.

The MCSS algorithm was developed based on the above quadrant search method and was effective in simulating multinomial classes in two horizontal dimensions [20]. The colocated Co-MCSS algorithm used in this paper is an extension of the random-path MCSS algorithm; therefore, their computation processes are similar. 

### 2.4. Transiogram Modeling and Cross-Field Transition Probability Matrix

To perform simulations using Co-MCSS, transiogram models are needed to provide transition probability values at any needed lag distances. The transiogram was formally established in recent years to meet the needs of related Markov chain models [18]. The initial purpose of proposing such a spatial correlation measure was to provide a practical way to estimate multistep transition probabilities from sparse point sample data [30]. Later, it was found that the transiogram could also be an excellent independent spatial measure to characterize the spatial variability of categorical spatial variables [18]. This spatial measure is related to some pioneer studies [31–35], which used or explored transition probability curves in some special conditions. There are different ways to get continuous transiogram models [36]. One is using nonparametric methods such as linear interpolation to interpolate experimental transiograms into continuous models. The second is using parametric methods (i.e., mathematical models) to fit experimental transiograms. Because the latter is relatively tedious and the sample data for soil map updating are usually sufficient for estimating reliable experimental transiograms, the first approach was chosen in this study. 

For a colocated cosimulation conditioned on one auxiliary variable, one cross-field transition probability matrix (CTPM) is sufficient. Transition probabilities in a CTPM can be estimated by counting point-to-point frequencies of different class pairs from the sample data of the primary variable to the colocated data of the auxiliary variable using the following equation:

(10) $$\begin{array}{c} b_{ik}=\frac{f_{ik}}{\sum_{j=1}^{n}f_{ij}}, \end{array}$$

where *f*_*ik*_ represents the frequency of transitions from class *i* of the primary variable to class *k* of the auxiliary variable and *n* is the number of classes of the auxiliary variable.

## 3. Case Study for Method Testing

### 3.1. Data, Parameters, and Outputs

The major purpose of this case study was to test the method proposed in this paper, rather than a real application. Because a real field soil survey was unavailable to us, synthetic data extracted from a piece of a real soil series map (9 km^2^ area) [20] was used in this case study. However, the spatial pattern and spatial relationships among the soil series can mimic some real-world situation, thus still providing an effective test to the proposed spatial statistical method. 

**Figure 3:**
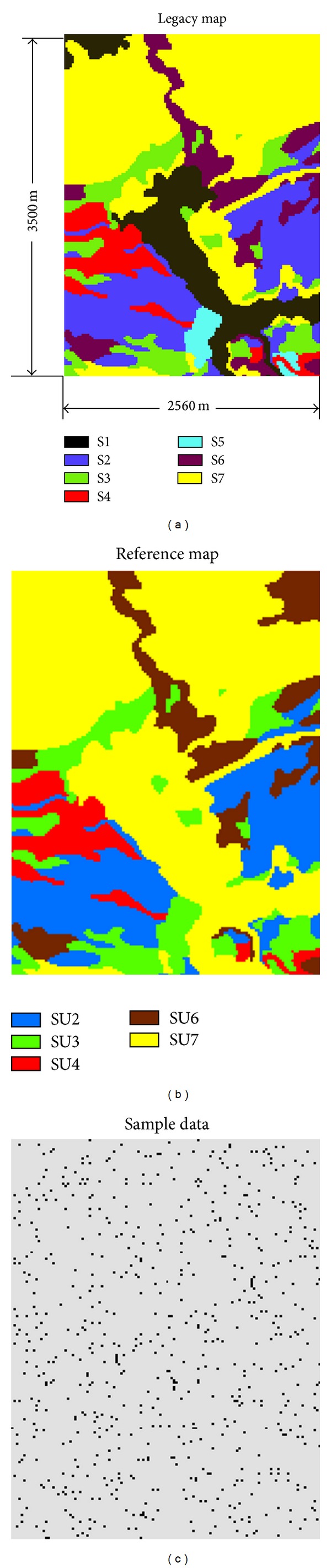
The data for categorical soil map update by Markov chain cosimulation: (a) the legacy soil map; (b) the reference soil map, representing the current distribution of soil series; (c) the sample data set (646 points), including field survey data and pseudosample data directly extracted from the unchanged areas in the legacy soil map. Previous soil series: S1, S2, S3, S4, S5, S6, and S7. Updated soil series: SU2, SU3, SU4, SU6, and SU7. SU2 = S2, SU3 = S3 + S5, SU4 = S4, SU6 = S6 + part of S7, and SU7 = S7 + S1 + part of S6.

The area was discretized into a 175 × 128 grid of 22,400 pixels, with a square pixel area of 400 m^2^. The soil map has seven soil types. Here, the exact soil series names are not our concern. For convenience, we denote them as S1, S2, S3, S4, S5, S6, and S7. This soil series map (Figure 3(a)) served as the legacy soil map for this study. The soil survey for delineating the legacy soil map was mainly done in the 1950s [37]. After five decades, such a soil map is likely outdated and would be improved by revision. We assumed that the legacy soil map from USDA was made with high-quality data at the mapping time, but that is now inaccurate. We further assumed that only a few of small areas in the legacy soil map were subject to soil type changes. For testing the suggested soil map update method, we designed the following soil series changes in the study area: S5 is joined to S3; S1 is joined to S7; part of S6 became S7 at the bottom middle east; and part of S7 became S6 at the top-right corner. As a result, we have five new soil series: SU2 (i.e., S2), SU3 (i.e., S3 + S5), SU4 (i.e., S4), SU6 (i.e., S6 + part of S7), and SU7 (i.e., S7 + S1  + part of S6). Soil series of S2, S3, and S4 were assumed to have no changes confirmed in the updated survey. The resulting new soil series distribution map (Figure 3(b)) was used as the reference soil map for checking simulated results.

Because we assumed only a few of small areas were subject to soil type changes, our limited field survey was also confined to these small areas. Thus, the survey data are insufficient and also biased for estimating the parameters (e.g., transiogram models) used in the cosimulation. Our suggestion is to use pseudosample data, that is, sample data directly extracted from unchanged areas in the legacy soil map. Therefore, we sampled a sparse data set of 646 points (Figure 3(c)) from the reference soil map, which cover both the changed and unchanged areas. These samples are randomly distributed, not purposively arranged with respect to soil type changes. Using this data set, we examined simulated results for other points to see how well our suggested method predicted soil type characteristics, both those that were unchanged and changed compared to the legacy map. The rationalities behind the sample data are that (1) for areas where soil types have changed, a field survey or visual observation through remote sensing is necessary to identify the changes on the map, and both methods may produce survey sample data for the update; and (2) for areas where soil types did not change, no matter how the judgment is made (from a field survey, remote sensing, or expertise), pseudosample data may be simply extracted from the legacy soil map. Pseudosample data extraction from a legacy map or from the combination of a legacy map and remotely sensed imagery can be carried out through human-computer interactions. Thus, it is not difficult to obtain sufficient sample data with a limited soil survey (i.e., a small set of real soil survey data). 

**Figure 4:**
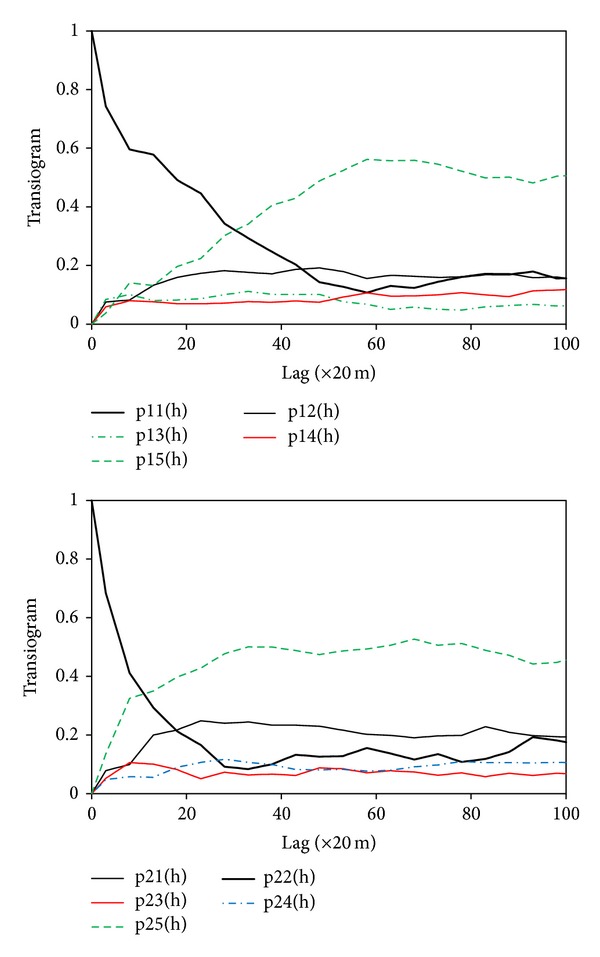
Two subsets of transiogram models interpolated from experimental transiograms estimated from the sample data. The numbers in transiogram labels (1 to 5) refer to the five updated soil series (i.e., SU2, SU3, SU4, SU6, and SU7), respectively.

Experimental transiograms were estimated from the sample data to generate transiogram models for conditional simulations. Two subsets of omnidirectional transiogram models interpolated from the experimental transiograms are provided in Figure 4 and show that cross-transiogram models have very different sills, related to their tail class proportions. Anisotropy was not considered because no identifiable anisotropic direction can characterize all soil types in the whole area while partial anisotropy is difficult to account for. The CTPM from the sample data set to the legacy soil map data is provided in Table 1. The numbers of columns and rows in the CTPM can be different and the classes in columns and rows need not have the same physical meanings, as they represent two different categorical variables, respectively. But for each row the transition probability values still sum to unity. Such a CTPM was used to express the cross-correlations between sample data and the legacy map. The sample data set has five soil types while the legacy soil map has seven soil types; thus, they have five types in common. These five soil types show strong cross-field autocorrelations, and two of them have no changes (i.e., cross-field transition probabilities are 1.0).

The search radius chosen is 30 pixels (i.e., 600 m). One hundred realizations were generated for the cosimulation conditioned on both the sample data and the legacy soil map using Co-MCSS, and occurrence probability maps were estimated from those realizations. The optimal prediction map was obtained from maximum occurrence probabilities. For the purpose of comparison, the same was done without conditioning on the legacy soil map using MCSS. The PCC (percentage of correctly classified locations) values were estimated for the optimal prediction map and realization maps against the reference soil map (sample data being excluded) to verify the simulation accuracies. 

### 3.2. Results of Cosimulation

**Figure 5:**
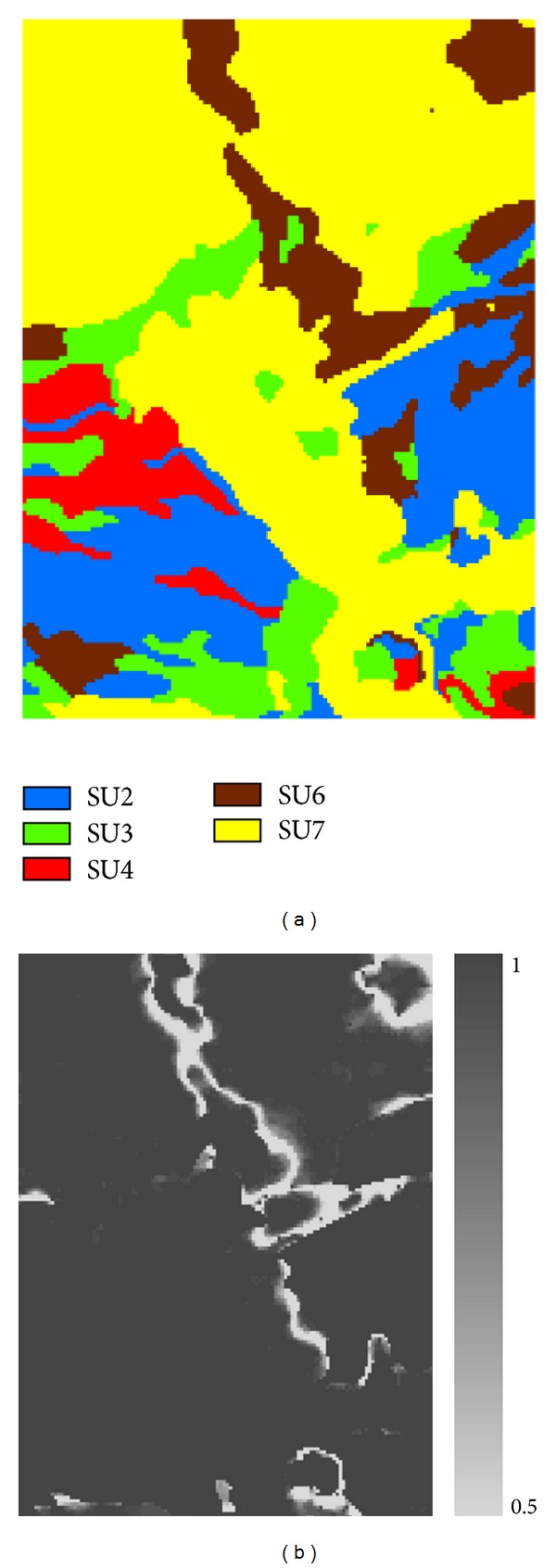
The optimal prediction map (a) and the maximum occurrence probability map (b) of updated soil series conditioned on sample data and the legacy soil map using the Co-MCSS method.

The updated categorical soil maps include the optimal prediction map, a series of simulated realization maps, and occurrence probability maps. But the most important should be the optimal prediction map generated from maximum occurrence probabilities that reflect the best predictions for a chosen method and available data. The optimal prediction map of the soil series and the corresponding maximum occurrence probability map (Figure 5) were estimated from simulated realizations generated by Co-MCSS, conditioned on both the sample data and the legacy soil map. The maximum occurrence probability map reflects the uncertainty of the optimal prediction map against the conditioning data. Comparing with the legacy soil map and the reference map (Figure 3) shows that the unchanged S2 and S4 were exactly reproduced as SU2 and SU4, respectively, and that the S3, which was merged with a minor soil series (S5) without other changes, was also exactly reproduced as SU3 in the optimal prediction map (Figure 5(a)). However, the S6 and S7, which changed into each other in some areas, were only approximately captured (as SU6 and SU7, resp.) with apparent uncertainty (see shallow gray areas in Figure 5(b)). The uncertainty mainly occurred at the boundary zones between these two soil series. Those areas of these two soil series that are located far away from each other were also well reproduced. Although soil type changes were confirmed by sample data only in two small areas (i.e., the top-left corner and the bottom-middle east) for S6 and S7, such changes caused the uncertainty of these two soil types in other areas in the updated map. This is reasonable because if a soil series is found to have changed at some places, it is quite possible that it may also have changed at other places, even if the changes at other places were not verified by the new survey. The changed areas of S6 and S7 (i.e., the top-left corner and the bottom-middle east) were well captured in the optimal prediction map (Figures 3(a) and 3(b)). The merging of S1 into S7 only increased the total area of SU7 and did not affect its uncertainty caused by the transformation in some areas between S6 and S7. 

**Figure 6:**
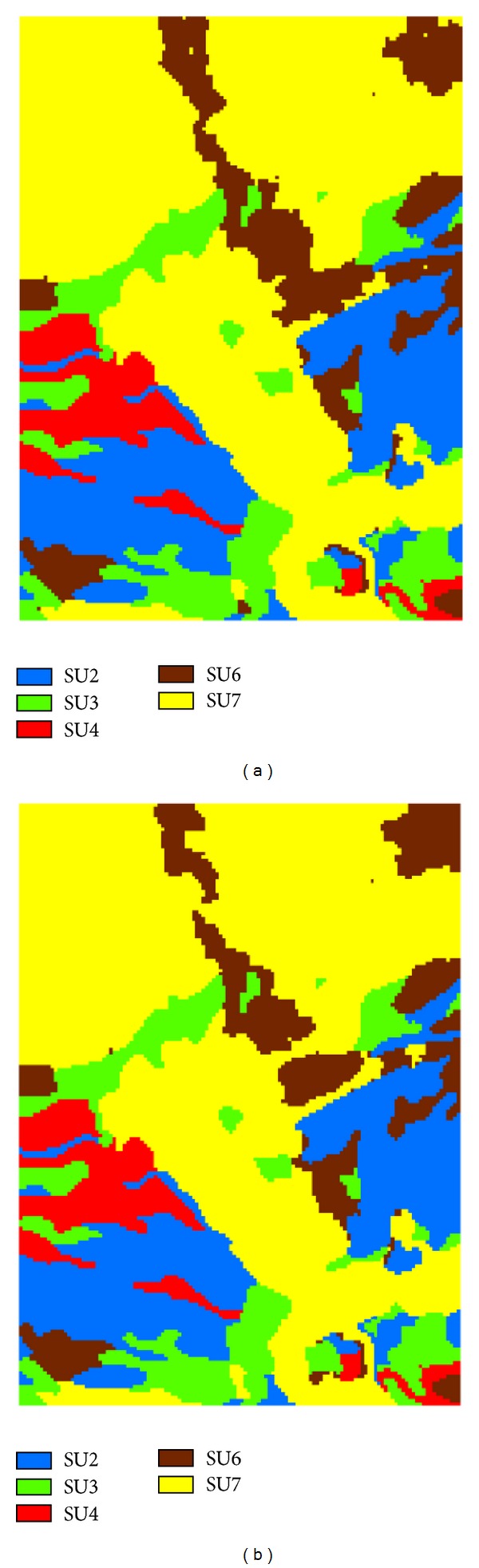
Two simulated realization maps of updated soil series conditioned on sample data and the legacy soil map using the Co-MCSS method.

Similar to hand-delineated maps, optimal prediction maps of categorical spatial variables normally also have an omission effect: minor classes are underrepresented because of their lower occurrence probabilities at most unsampled locations and major classes are consequently overrepresented [20, 38]. This situation also occurred on the predicted soil maps using the MLR method [3]. Because of the contribution of the legacy soil map and the lack of apparent minor classes, this effect is visually not obvious in the optimal prediction map by Co-MCSS compared with the simulated realization maps (Figure 6), which normally do not have such an effect.

**Figure 7:**
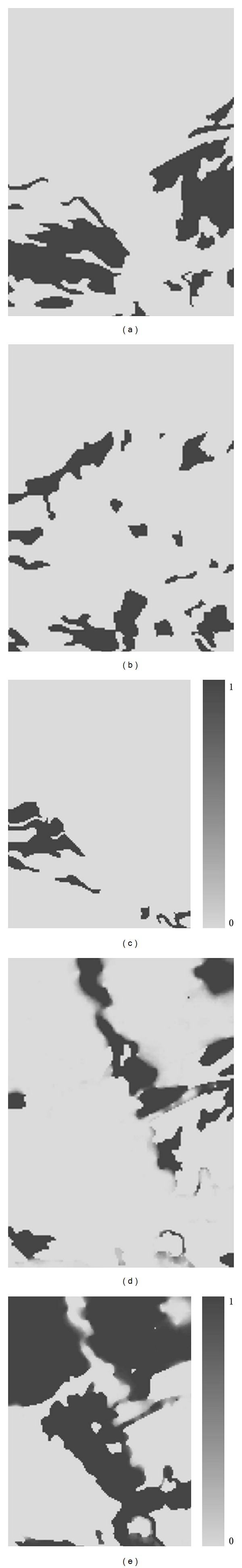
Occurrence probability maps of updated single soil series conditioned on the sample data and the legacy soil map using the Co-MCSS method. (a) SU2; (b) SU3; (c) SU4; (d) SU6; and (e) SU7.

The simulated realization maps (Figure 6) and occurrence probability maps of single soil series (Figure 7) further verify the judgments based on the results provided in Figure 5. Between those two realizations (Figure 6), soil series SU2, SU3, and SU4 do not show differences in pattern details, but some differences for SU6 and SU7 do exist. In Figure 7, occurrence probability maps of SU2, SU3, and SU4 are simply binary maps (i.e., 0 and 1), meaning that they are simply inherited from the legacy map because no changes other than taxonomic adjustments were confirmed by sample data. This does not mean that these soil series in the updated soil map are correct. They just made no changes against the legacy map. Uncertainties in the occurrence probability maps of SU6 and SU7 are clear but mainly appear along the boundaries between them. 

### 3.3. Comparison with MCSS

**Figure 8:**
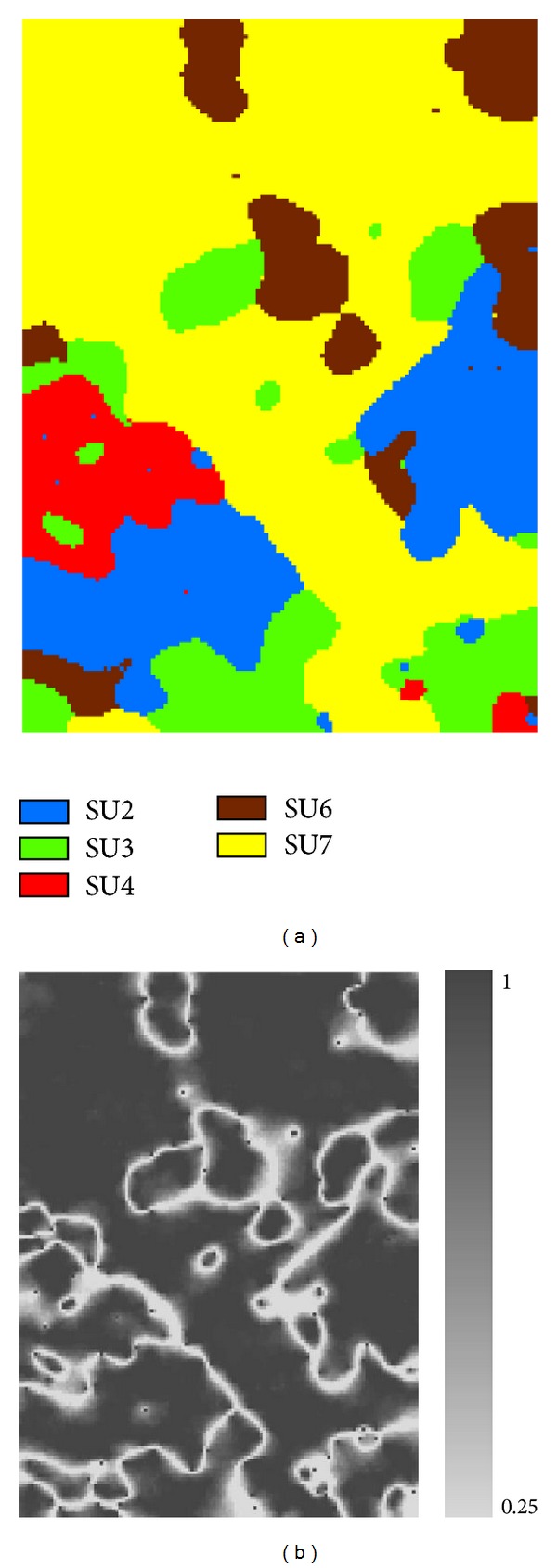
The optimal prediction map (a) and the maximum occurrence probability map (b) of updated soil series conditioned only on the sample data using the MCSS method.

To verify the improvement and advantages of Co-MCSS over MCSS, which cannot incorporate auxiliary information, we also used the MCSS method to conduct a simulation conditioned on the same sample data. Comparing optimal prediction and maximum occurrence probability maps (Figure 8) generated by MCSS to those generated by Co-MCSS (see Figure 5) clearly indicates distinct differences: (1) unlike MCSS, Co-MCSS can capture pattern details, particularly linear features; and (2) MCSS generated much more uncertainty than did Co-MCSS. Thus, the contribution of the legacy soil map to the accuracy of the simulated results by Co-MCSS is huge due to the assumed good quality of the legacy map. The simulated realization maps and occurrence probability maps of single soil series by MCSS are omitted (one may see similar simulations in [20]) but corroborated the same conclusions. 

The PCC value represents the accuracy of a classified map compared to reference data. Using the reference map modified from the legacy soil map (Figure 3(c)), we calculated the PCC values of the optimal prediction maps and the averaged PCC values of the simulated realization maps generated by Co-MCSS and MCSS. The results (Table 2) show that the optimal prediction map and the simulated realization maps by Co-MCSS have a substantive improvement in simulation accuracy over those by MCSS. For the optimal prediction map, the improvement is about 16% in absolute values and about 19% in relative values, whereas for the realization maps, the improvement is about 18% in absolute values and about 23% in relative values. These accuracy improvements are attributed to the legacy soil map as auxiliary information. The accuracies of the optimal prediction map and the realization maps by Co-MCSS are generally above 97%. Such a high accuracy should be related to the relatively small soil changes. If the soil series in the study area have large changes since last survey or if the legacy soil map is of lower quality with many errors confirmed, the legacy map cannot contribute so much in improving simulation accuracy. Of course, unidentified errors in the legacy map will be brought into the updated soil map invisibly.

Sample data directly extracted from the unchanged areas of the legacy soil map are not real survey data for map updating. They were used for fairly estimating the transiogram models and the cross-field transition probability parameters and also for conditioning the simulations. This study does not show that the conditioning of the extracted pseudosample data for unchanged soil series (including merged unchanged soil series) in simulations is necessary, as these unchanged soil series are simply reproduced from the legacy soil map. But if a soil type change is confirmed at a place by a survey sample datum, pseudosample data should not be extracted nearby unless they are surely correct because pseudosample data confirm the unchanged status of soil series at their locations.

## 4. Conclusions

Updating categorical soil maps is necessary for many reasons, such as being outdated or of low quality. We assumed that the most recent legacy soil maps may need only limited corrections due to modest natural and anthropogenic soil changes occurring during the intervening time period. As a result, updates to the legacy maps are necessary in only the changed and mistakenly mapped areas. In essence, we assume that the legacy soil maps were outdated but of good quality. Such a situation may be applicable to the soil map update of the United States, where quite detailed large-scale categorical soil maps exist for each county in most states. 

We introduced the random-path Co-MCSS algorithm, which extended the random-path MCSS algorithm, for revising categorical soil maps and applied it to a case study of synthetic data that involved the revision of a legacy soil series map using limited survey data. Simulated results show that (1) Co-MCSS can greatly improve simulation accuracy of soil types via the contribution of legacy soil maps, and (2) the accuracy of the optimally predicted soil map by Co-MCSS is better than that by MCSS, at least in a situation characterized by the use of limited survey sample data. Co-MCSS demonstrated the following merits: (1) if a soil type has no changes confirmed in an update survey or if it is decided to be reclassified into another type that is deemed to have no change, it will be simply reproduced in updated soil maps; (2) if a soil type has changes in some areas (e.g., an update survey confirmed the changes or map examination found previous wrong classification), it will be simulated with uncertainty; (3) if a soil type has no change in an area but has changes in other distant areas, it still can be captured with little changes in the area. The occurrence probability maps estimated from the simulated realizations reflect only the uncertainty verified by new survey sample data and do not reflect the uncertainty contained in the legacy soil map but unverified by sample data. In general, we conclude that Co-MCSS may provide a practical spatial statistical tool for revising categorical soil maps. 

Finally, other related data, such as land cover/land use and discretized DEM-derived data (e.g., elevation), are often correlated with the spatial distributions of soil series and may also be incorporated as auxiliary information to improve the accuracy of soil mapping, especially when legacy soil maps are of low quality or unavailable and the survey sample data are very sparse. In this study, because we assumed that legacy soil maps were available and of high quality and only limited soil changes occurred, other auxiliary variables were not considered. 

## Figures and Tables

**Table 1:** Cross-field transition probability matrix from sample data (5 soil series) to colocated data in the legacy soil map (7 soil series).

Data	Soil series^†^	Legacy soil map						
		S1	S2	S3	S4	S5	S6	S7
Sample data	SU2	.0000	1.0000	.0000	.0000	.0000	.0000	.0000
	SU3	.0000	.0000	.9011	.0000	.0989	.0000	.0000
	SU4	.0000	.0000	.0000	1.0000	.0000	.0000	.0000
	SU6	.0000	.0000	.0000	.0000	.0000	.8143	.1857
	SU7	.2169	.0000	.0000	.0000	.0000	.0271	.7560

^†^S1 is a soil series in the legacy soil map. SU2 is a soil series in the updated soil map.

**Table 2:** Percentages of correctly classified locations (PCCs) of optimal prediction maps and simulated realizations (averaged from 100 realizations) generated by Co-MCSS and MCSS. PCCs (%) are estimated relative to the reference soil map with sample data being excluded.

Item	Accuracy	
	Optimal prediction map	Realization maps
MCSS	82.50	79.32
Co-MCSS	98.25	97.23
Absolute improvement^†^	15.75	17.91
Relative improvement^‡^	19.09	22.58

^†^Absolute improvement = PCC of Co-MCSS − PCC of MCSS. ^‡^Relative improvement = absolute improvement/PCC of MCSS × 100.
